# Endoplasmic Reticulum Stress and Unfolded Protein Response Signaling in Plants

**DOI:** 10.3390/ijms23020828

**Published:** 2022-01-13

**Authors:** Hakim Manghwar, Jianming Li

**Affiliations:** 1 State Key Laboratory for Conservation and Utilization of Subtropical Agro-Bioresources, College of Forestry and Landscape Architecture, South China Agricultural University, Guangzhou 510642, China; hakim@scau.edu.cn; 2 Guangdong Key Laboratory for Innovative Development and Utilization of Forest Plant Germplasm, College of Forestry and Landscape Architecture, South China Agricultural University, Guangzhou 510642, China; 3 Department of Molecular, Cellular and Developmental Biology, University of Michigan, Ann Arbor, MI 48109, USA

**Keywords:** plants, ER, ER stress, UPR, IRE1, bZIP17, bZIP28, bZIP60

## Abstract

Plants are sensitive to a variety of stresses that cause various diseases throughout their life cycle. However, they have the ability to cope with these stresses using different defense mechanisms. The endoplasmic reticulum (ER) is an important subcellular organelle, primarily recognized as a checkpoint for protein folding. It plays an essential role in ensuring the proper folding and maturation of newly secreted and transmembrane proteins. Different processes are activated when around one-third of newly synthesized proteins enter the ER in the eukaryote cells, such as glycosylation, folding, and/or the assembling of these proteins into protein complexes. However, protein folding in the ER is an error-prone process whereby various stresses easily interfere, leading to the accumulation of unfolded/misfolded proteins and causing ER stress. The unfolded protein response (UPR) is a process that involves sensing ER stress. Many strategies have been developed to reduce ER stress, such as UPR, ER-associated degradation (ERAD), and autophagy. Here, we discuss the ER, ER stress, UPR signaling and various strategies for reducing ER stress in plants. In addition, the UPR signaling in plant development and different stresses have been discussed.

## 1. Introduction

Plants are becoming exposed to numerous environmental changes during their lifecycle and use complex integrated mechanisms to sense and adapt to these conditions for their growth and development [1,2]. Plants have evolved a number of strategies to respond to various types of stresses at diverse levels, from gene expression alterations to changes in morphology [3,4,5]. The sensing and transduction of environmental signals have been extensively studied in stressed plants, revealing potential strategies for improving agricultural productivity and plant tolerance against different stresses [6]. The environmental sensors ultimately induce changes in metabolic pathways, protein synthesis, and gene expression to enhance plant tolerance against various stresses [6]. This review paper discusses the ER and its functions, the ER stress, and different strategies that play a crucial role in reducing ER stress in plants. Moreover, the role of the unfolded protein response (UPR) signaling in plant development and in various stresses has been discussed.

## 2. Endoplasmic Reticulum (ER)

**Figure 1:**
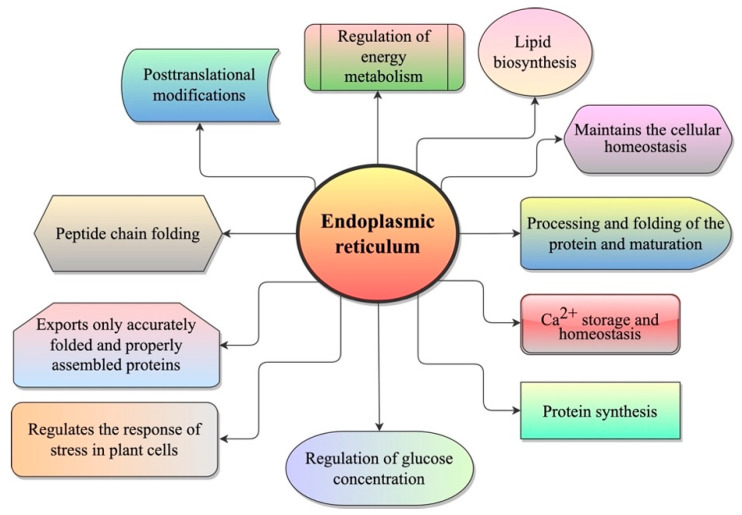
Different functions of the ER in plants.

ER is a large, complex, and highly dynamic cytoplasmic membrane system of eukaryotic cells and is considered to be a central network of interconnected tubules and flattened cisternae that extend across the cytoplasm [7,8]. The ER network occupies a significant portion of the cytoplasm with its membrane, accounting for ~50% of total cellular membranes [9]. ER plays a crucial role in protein synthesis, peptide chain folding and processing, post-translational modifications, lipid biosynthesis, Ca^2+^ storage and homeostasis, and the regulation of glucose concentration [10,11] (Figure 1). This organelle provides an oxidative environment to facilitate the formation of a disulfide bond and is loaded with molecular chaperones [12]. In addition, the ER is involved in regulating the stress responses in animal and plant cells [13].

## 3. ER Stress

ER plays a vital role in maintaining cellular homeostasis in different cellular processes through its functions, such as initial modification and folding of transmembrane and secretory proteins. Endogenous factors and environmental conditions can increase the demand for protein folding machinery. Many factors induce ER stress in plants, such as pathogens, environmental stresses, salinity, and drought, resulting in a higher load on secretory proteins in the ER [14,15]. These stresses accumulate misfolded or unfolded proteins that induce ER homeostasis imbalance, which is called ER stress [16,17,18]. Protein synthesis and modification lead to errors in almost one-third of the nascent proteins in the ER [19,20,21]. ER stress often leads to growth retardation in Arabidopsis (*Arabidopsis thaliana*) [22]. To overcome ER stress, the stress sensors localized in the ER activate distinct downstream organelle-nucleus signaling pathways to invoke a cytoprotective response, which is known as UPR [23]. UPR has recently been recognized as an important intracellular signaling pathway for linking ER proteostasis with gene regulation in the nucleus to reduce ER stress [6]. Although the molecular mechanism of ER stress in plants is not as well understood as it is in animals [24]. The expansion and abundance of genes related to ER stress, revealed by the genome sequencing of various plant species, suggest that plants use more ER stress responses than animals in order to adapt to the environment [24].

## 4. Chemical Inducers for the Accumulation of the Unfolded Protein

### 4.1. Tunicamycin (TM) Stress

TM and Dithiothreitol (DTT) have been found to induce UPR by interfering with protein folding in the ER [25,26]. TM triggers ER-mediated stress in various eukaryotic species, such as plants, yeast, and humans [27,28,29,30]. TM inhibits the *N*-linked glycosylation (*N*-glycans formation) by interfering with the GlcNac phosphotransferase enzyme, which is responsible for the initial glycosylation steps [31]. The *N*-glycans are essential for the proper folding and stability of proteins, as well as their transportation to the Golgi apparatus for the packaging of vesicles and secretion. Besides, *N*-glycans are involved in the post-translational alteration of microbe-associated molecular patterns (MAMPs) receptors and immune response in plants [30,32,33].

### 4.2. Dithiothreitol (DTT) Stress

DTT is a powerful reducing agent that induces acute ER stress by disrupting the redox conditions required to form disulfide bridges in proteins [34,35]. DTT is a robust reducing agent commonly used to promote reductive pressure. It can cross membranes and inhibit the formation of a disulfide bond. DTT treatment induces reductive stress, leading to the accumulation of misfolded proteins in the ER [36,37]. However, as a strong inducer of ER stress, DTT has not been considered as an ideal UPR triggering agent for in vivo studies because it can inhibit disulfide bond formation in the ER and cytosol during protein formation, making it unspecific to ER stress [38]. Besides, TM and DTT have differential effects on ER stress kinetics and can influence the expression of UPR target genes [30,39]. Arabidopsis basic leucine zipper 28 (bZIP28), an ER membrane-associated transcription factor (TF) (ER membrane-associated basic leucine zipper), was triggered by an ER stress response induced by exposure to DTT and TM or adverse environmental conditions [37,40]. In addition, TM or DTT may chemically induce basic leucine zipper TF 60 (bZIP60) splicing [41,42].

## 5. UPR Signaling in Plant Development

UPR has been broadly studied in the context of ER stress, although recently more attention has been diverted to the role of UPR in plant development and defense. UPR has been found to play a crucial role in both reproductive and vegetative development [14]. Additionally, UPR plays an unexpected role in hormone biology, which may explain the effect of UPR on vegetative growth and development [14]. Normal plant growth and development require UPR. Normal growth and development require the mobilization of basic leucine zipper 17 (bZIP17) into the nucleus [43]. The triple mutant inositol-requiring enzyme 1 (IRE1a IRE1b) bZIP17 grew abnormally under normal growth conditions and was also defective in the stress signaling pathways. The functions of bZIP17 in *A. thaliana* development were observed in another study using genomic and genetic approaches. In contrast to bZIP28 and bZIP60, the bZIP17 is not a primary UPR activator, but works in conjunction with bZIP28 to regulate development-related genes, particularly stress maintenance and root elongation [44]. In *A. thaliana*, ER stress induces the expression of *NAC103*. *NAC103* overexpression has pleiotropic effects on plant growth, plays a vital role in inducing the expression of some UPR downstream genes under normal growth conditions [45]. BLISTER (BLI) protein is localized to the Golgi, which negatively regulates IRE1a/IRE1b activity under normal growth conditions. A *BLI* loss-of-function mutation results in prolonged up-regulation of non-canonical UPR downstream genes and canonical UPR genes, resulting in growth retardation and cell death [46]. *A. thaliana* aquaporins; SIP1;1, SIP1;2, and SIP2;1 are localized in the ER. The aquaporin SIP2;1 plays an important role in alleviating ER stress. A reduction in the elongation of the pollen tube and pollen germination was observed in the absence of *SIP2;1* [47]. The basal mRNA level of binding protein 3 (*BiP3*) is an essential ER stress-induced gene in pollen, suggesting that pollen has experienced ER stress under normal growth conditions [47]. Plants lacking SQUAMOSA PROMOTER BINDING PROTEIN-LIKE 6 (SPL6) showed hyperactivation of IRE1, leading to cell death in rice panicles, which indicates that the SPL6 is an important survival factor in suppressing persistent or extreme ER stress conditions [48]. DERLIN-like protein (OsDER1) is a homolog of yeast and mammal DER1 localized in the ER and has been observed to be accumulated considerably in rice under ER stress. Overexpression or suppression of *OsDER1* leads to the activation of UPR and hypersensitivity to the ER stress, and suppression results in shrunken and floury seeds [49].

## 6. UPR Signaling in Different Stresses

**Figure 2:**
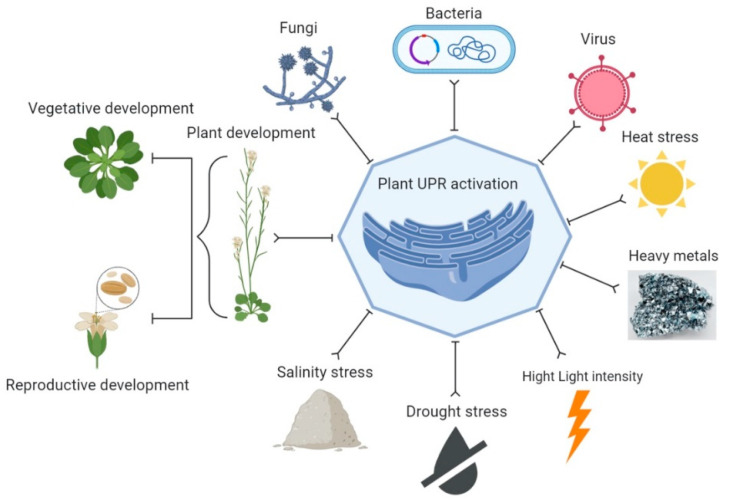
Activation of the UPR. A variety of stresses (biotic and abiotic) and plant development processes (vegetative and reproductive) trigger the UPR by excessive accumulation of unfolded proteins in the ER or cause an imbalance in the supply of amino acids, which leads to the activation of one or more UPR arms. This figure was created by using BioRender software.

The UPR can be activated by various stresses in plants that induce the accumulation of unfolded proteins in the ER lumen [50,51] (Figure 2). UPR has been involved in the immunity and development of plants and provides defense against different stresses [14,42,44], such as heat [52], drought [53], salinity [54,55], osmotic pressure, high light intensity and heavy metals. These stresses disturb protein folding [15,56]. In addition, UPR is activated under protein synthesis overload conditions when the need for protein folding simply does not meet demands [57,58]. However, cells commit to programmed cell death (PCD) during the failure of UPR in chronic or unresolved ER stress conditions [23,59,60,61]. Biotic agents have more complex effects on the UPR. Various biotic agents have been reported to induce the UPR or require the UPR to infect plants. A study observed that when *Nicotiana benthamiana* was inoculated with the host-pathogen and non-host pathogens, *Pseudomonas syringae* and *Pseudomonas cichorii*, respectively, the host pathogen *P. syringae* did not induce the expression of bZIP60. At the same time, the expression of bZIP60P was induced by the non-host pathogen, *P. cichorii*. However, the plants became more susceptible to *P. syringae* when virus-induced gene silencing (VIGS) was used to silence the bZIP60 in *N. benthamiana* [62]. UPR is induced and required by various plant viruses for successful infection. In another study, a knockout in bZIP60 was observed to suppress the viral symptoms and the transgenic expression of an activated form of bZIP60 that could suppress the symptoms in the bZIP60 knockout [63]. Various genes are associated with the ER stress response by multiple stresses and plant development in *A. thaliana* and many other major crops/plants (Table 1).

Many studies in rice (*Oryza sativa* L.) (*OsbZIP50*), maize (*Zea mays* L.) [41] and *A. thaliana* [64,65] have reported IRE1 splicing of the heat-induced *bZIP60*. Furthermore, HRD3A is an influential part of plant ERAD and plays a crucial role in plant UPR. In *A. thaliana*, a defect in HRD3A results in UPR alteration, increased sensitivity of the plant to salt, and the retention of ERAD substrates in plant cells [66]. The stress-induced and ER membrane-localized functional ubiquitin conjugation enzyme (E2) UBC32 connects the process of ERAD and brassinosteroid (BR)-mediated growth promotion and salt stress tolerance in *A. thaliana*. UBC32 affects the stability of barley powdery mildew O (MLO) mutant MLO-12, a known ERAD substrate [67]. Arabidopsis homolog (AtOS9) of an ER luminal lectin Yos9 plays a vital role in recognizing a unique asparagine-linked glycan on misfolded proteins. AtOS9 is a glycoprotein localized to the ER and co-expressed with various predicted/known ER chaperones [68]. *Arabidopsis ethyl methane sulfonate-mutagenized brassinosteroid insensitive 1 suppressor 7 (EBS7)* gene encodes an ERAD component localized in the ER membrane. The accumulation of EBS7 has been observed under ER stress, and its mutations cause hypersensitivity to salt and ER stresses [69]. Arabidopsis ERAD genes, *HRD1A*/*1B*, and *CER9* might regulate the heat stress response. HRD1A/1B and CER9 collaboratively regulate plant thermos tolerance and the expression of both UPR and Cytosolic Protein Response (CPR) genes, no matter under heat stress or normal conditions [70]. WRKY75 is an ER-stress cellular response regulator as its expression directly responds to ER stress-inducing chemicals, such as TM and DTT. Plants that express *WRKY75* show tolerance to salt stress, connecting ER and abiotic stress responses [71].

The overexpression of *BhbZIP60*, an *AtbZIP60* homologous from the *Boea hygrometrica* plant, has resulted in increased resistance to mannitol and drought stresses [72]. Expression profile analyses of soybean plants treated with an osmotic stress inducer (polyethylene glycol) or ER stress inducers (TM/azidothymidine) indicate a correlation between the osmotic stress pathway and ER stress [73]. *BiP* expression is up-regulated in wheat (Triticum aestivum L.) during osmotic stress-related cell death [74]. The MfSTMIR, which encodes a highly conserved ER-membrane-localized RING E3 ligase in leguminous plants, plays an essential role in combatting salt and ER stress in Medicago. In another study, the expression of MfSTMIR was found to be induced by TM and salt. The mtstmir loss-of-function mutants showed impaired induction of BiP1/2 and BiP3 ER stress-responsive genes under TM treatment and sensitivity to salt stress [75].

In *A. thaliana*, the overexpression of bZIP60 improved tolerance against salt stress [76]. Xi et al. showed that SAL1 loss-of-function caused improved Cd tolerance and reduced ER stress in *A. thaliana* [77]. Arabidopsis Golgi anti-apoptotic proteins 1 and 3 (GAAP1, 3) were observed to resist PCD against ER stress and negatively modulate the IRE1-bZIP60 pathway. Mutations in *GAAP1*/*GAAP3* or/and Membrane-associated progesterone receptor 3 (*MAPR3*) increase the vulnerability of seedlings to ER stress [78]. Moreover, GAAP1 and GAAP3 are involved in regulating cell death and UPR. GAAP1 to GAAP3 were observed to play redundant roles in delaying the UPR activation induced by ER stress and inhibiting cell death [79].

Many phytohormones are involved in UPR signaling. Increasing evidence supports newly emerging roles for plant hormones, such as jasmonic acid (JA) [80], salicylic acid (SA) [81], auxin, and Ethylene (ETH) [80,82], secondary messengers (e.g., Ca^2+^) [83], as well as other signaling molecules such as Reactive oxygen species (ROS) and sugars, as essential regulators of the UPR in plants [84]. JA signaling pathway is involved in defense against necrotrophic pathogens. A study showed that transcriptional levels of chaperone protein genes, such as BiP, calreticulin (CRT), calnexin 1-like (CNX 1-like), and protein disulfide isomerase (PDI), and genes involved in the IRE1-bZIP60 pathway, were all significantly induced in *Nicotiana attenuata* leaves after the inoculation of *A. alternata*. The silencing of *bZIP60* or *IRE1* gene increased the susceptibility of *N. attenuata* plants to *A. alternata*. IRE1-bZIP60 pathway is needed for the resistance of *N. attenuata* to *A. alternata*, and JA signaling pathway plays an essential role in eliciting the IRE1-bZIP60 pathway and chaperone protein genes [85]. SA has been observed to play a crucial role in ER stress signaling and UPR regulation under stress conditions [24], although its mode of action is unknown. In a study, the relationship between ER stress and SA-mediated defense responses was postulated, and spatiotemporal change was described [86]. In another study, Wang et al. [87] discovered that SA-induced master regulator protein NPR1 (nonexpressor of pathogenesis-related (PR) genes 1) regulates numerous ER stress and UPR components induced by SA during systemic acquired resistance (SAR) development in *A. thaliana*.

**Table 1:** Involvement of different genes in ER and other stresses.

Gene	Function	Stress	Plant/Crop	Reference
*HRD3A*	Defects in *HRD3A* cause alteration in the UPR, increased plant sensitivity to salt, and retention of ERAD substrates in plant cells.	Salt and ER stresses	Arabidopsis (*A. thaliana*)	[66]
*UBC32*	*UBC32* affects the stability of barley powdery mildew O (MLO) mutant MLO-12, a known ERAD substrate.	ER stress	Arabidopsis (*A. thaliana*)	[67]
*AtOS9*	*AtOS9* is an ER-localized glycoprotein and co-expresses with various predicted/known ER chaperones.	ER stress	Arabidopsis (*A. thaliana*)	[68]
*NAC103*	ER stress induces the expression of *NAC103*. Overexpression of *NAC103* has pleiotropic effects on plant growth. It plays a crucial role in inducing the expression of some UPR downstream genes under normal growth conditions.	ER stress	Arabidopsis (*A. thaliana*)	[45]
*EBS7*	*Arabidopsis ethyl methane sulfonate-mutagenized brassinosteroid insensitive 1 suppressor 7 (EBS7)* gene observed to be accumulated under ER stress, and its mutations lead to hypersensitivity to salt and ER stresses.	ER and salt stresses	Arabidopsis (*A. thaliana*)	[69]
*WRKY75*	*WRKY75* is an ER-stress cellular response regulator. Plants expressing *WRKY75* show tolerance to salt stress, which connects the ER and abiotic stress responses.	ER and salt stresses	Arabidopsis (*A. thaliana*)	[71]
*AtNRP1*, *AtNRP2* and AtNRPs; (*ANAC036* and *gVPE*)	Loss-of-function of *AtNRP1* and *AtNRP2* attenuates the cell death caused by ER stress. Osmotic and ER stresses have been shown to induce AtNRPs; (*gVPE* and *ANAC036*).	ER stress	Arabidopsis (*A. thaliana*)	[88]
*AtHSPR*	*AtHSPR* (*A. thaliana* Heat Shock Protein Related) is involved in ER stress signaling and cell death caused by salt stress.	ER stress	Arabidopsis (*A. thaliana*)	[89]
*SAL1*	*SAL1* is a negative regulator of stress signaling and is linked to plant stress responses. Loss-of-function of *SAL1* resulted in a significant reduction in ER stress and a significant increase in Cd tolerance.	ER and cadmium (Cd) stresses	Arabidopsis (*A. thaliana*)	[77]
*HOP*	HSP70-HSP90 organizing protein (*HOP*) is a member of the cytosolic cochaperones family. *HOP3* interacts in vivo with cytosolic HSP70 and HSP90, and with binding immunoglobulin protein (BiP), an HSP70 protein is localized in the ER.	ER stress	Arabidopsis (*A. thaliana*)	[90]
*CER9* and *HRD1A*/*1B*	Arabidopsis ERAD genes, *HRD1A*/*1B* and *CER9* might regulate the heat stress response. *HRD1A*/*1B* and *CER9* collaboratively regulate plant thermos tolerance.	ER and heat stresses	Arabidopsis (*A. thaliana*)	[70]
*AtNTL7*	*AtNTL7* is a membrane-tethered NAC TF that leads to resistance to ER stress. Overexpression of *AtNTL7* exhibits strong resistance to ER stress.	ER stress	Arabidopsis (*A. thaliana*)	[91]
*HY5*	Mutation of a main light signaling component, ELONGATED HYPOCOTYL 5 (*HY5*), leads to ER stress tolerance. HY5 negatively regulates the UPR by competing with bZIP28 for binding to the G-box-like element present in the ER stress response element.	ER stress	Arabidopsis (*A. thaliana*)	[56]
*OsDER1*	Suppression or overexpression of *OsDER1* results in the activation of UPR and hypersensitivity to ER stress and suppression leads to shrunken and floury seeds.	ER stress	Rice (*O. sativa* L.)	[49]
*SPL6*	Mutation of SQUAMOSA PROMOTER-BINDING PROTEIN-LIKE 6 (*SPL6*) up-regulates the expression of IRE1 and persistent UPR, which causes cell death and the abortion of rice apical panicles.	ER stress	Rice (*O. sativa* L.)	[48]
*EMR*	ERAD-mediating RING finger protein (*EMR*) plays an essential role in the plant ERAD system, affecting the BR signaling under ER stress conditions.	ER stress	Arabidopsis (*A. thaliana*)	[92]
*GAAP1*	*GAAP1* (Arabidopsis Golgi anti-apoptotic protein 1) regulates the PCD and UPR. *GAAP1* prevents cell death induced by ER stress and encourages the recovery of plant growth by attenuating the UPR process mediated by IRE1 after ER stress relief.	ER stress	Arabidopsis (*A. thaliana*)	[78,79]
*BLI*	BLISTER (*BLI*) protein loss-of-function mutation up-regulates the canonical UPR of non-canonical UPR downstream genes, inducing growth retardation and cell death.	ER stress	Arabidopsis (*A. thaliana*)	[46]
*hyl1*	HYPONASTIC LEAVES1 (*hyl1*) mutant plants are more susceptible to TM, which causes ER stress.	ER stress	Arabidopsis (*A. thaliana*)	[93]
*FAD2*	The 7 fatty acid desaturases (*FADs*) desaturate each glycerolipid class differently in plastids and ER. FAD2 mutants have resulted in a hypersensitive response to TM through systematic screening of FAD mutants.	ER stress	Arabidopsis (*A. thaliana*)	[94]
*NF-YC14*	*NF-YC14* involves in regulating the ER stress response. *NF-YC14* overexpression improves plant tolerance to ER stress and increases the expression of downstream genes for ER stress response.	ER stress	Arabidopsis (*A. thaliana*)	[95]
*GAAP1*, *GAAP3*, and *MAPR3*	Arabidopsis Golgi anti-apoptotic proteins 1 and 3 (*GAAP1*, *3*) resist PCD against ER stress and negatively modulate the IRE1-bZIP60 pathway. Mutations in *GAAP1*/*GAAP3* or/and Membrane-associated progesterone receptor 3 (*MAPR3*) increase the vulnerability of seedlings to ER stress.	ER stress	Arabidopsis (*A. thaliana*)	[96]
*MfSTMIR*	*MfSTMIR* plays a crucial role in salt and ER stress response. The expression of *MfSTMIR* was observed to be induced by TM and salt.	ER and salt stresses	Sickle medic (*Medicago falcata*)	[75]
*SIP1;1*, *SIP1;2*	*A. thaliana* aquaporins; *SIP1;1*, *SIP1;2* and *SIP2;1* are localized in the ER. The aquaporin *SIP2;1* involves alleviating the ER stress. The absence of *SIP2;1* reduces pollen tube elongation and pollen germination.	ER stress	Arabidopsis (*A. thaliana*)	[47]
*BiP3*	The basal mRNA level of *BiP3* is an important gene induced by ER stress in pollen.	ER stress	Arabidopsis (*A. thaliana*)	[47]
*PAWH1* and *PAWH2*	*PAWH1* and *PAWH2* are localized in the ER membrane and associated with Hrd1 through EMS-mutagenized Bri1 Suppressor 7 (*EBS7*). Removal of two *PAWHs* constitutively triggers the UPR and compromises the resistance to stress.	ER stress	Arabidopsis (*A. thaliana*)	[97]
*AtOTU1*	*AtOTU1* selectively hydrolyzes various forms of ubiquitin chains. *AtOTU1* is required to process plant ERAD substrates.	ER stress	Arabidopsis (*A. thaliana*)	[98]
*AtSec62*	Arabidopsis Sec62 (*AtSec62*) is required for plant development and may function as an ER-phagy receptor in plants.	ER stress	Arabidopsis (*A. thaliana*)	[99]
*TIN1*	Transcriptional induction of Tunicamycin induced 1 (*TIN1*) by ER stress was partially regulated by AtbZIP60. The accumulation of *TIN1* protein was observed in response to TM treatment.	ER stress	Arabidopsis (*A. thaliana*)	[28]

## 7. Strategies to Reduce ER Stress

Many strategies have been used to reduce ER stress, including the UPR, ER-associated degradation (ERAD), and autophagy.

### 7.1. Unfolded Protein Response (UPR)

UPR is an intracellular signaling mechanism activated by ER stress and has been designed to restore the ER function and to ignite the PCD processes when ER stress remains unresolved [100]. ER stress leads to the accumulation and aggregation of the unfolded proteins in the ER lumen. Moreover, UPR originates at the ER, where it overcomes the ER stress, restores the ER homeostasis, and leads to the ER chaperones and foldases synthesis to attenuate the ER stress [95,101]. These ER chaperones and foldases are BiP, protein disulfide isomerase (PDI), glucose-regulated protein (GRP94), peptidyl-prolyl isomerases (PPI) or immunophilins, calnexin and calreticulin [102]. Both signaling pathways eventually result in the upregulation of genes to either correctly fold or degrade misfolded proteins and regulate translation and transcription for restoring the ER homeostasis [28,103]. UPR may relieve the transient ER stress, whereas persistent ER stress can result in PCD [104,105].

### 7.2. Mechanism of UPR Signaling Pathway in Plants

#### 7.2.1. Regulated IRE-1 Dependent Splicing (RIDS)

**Figure 3:**
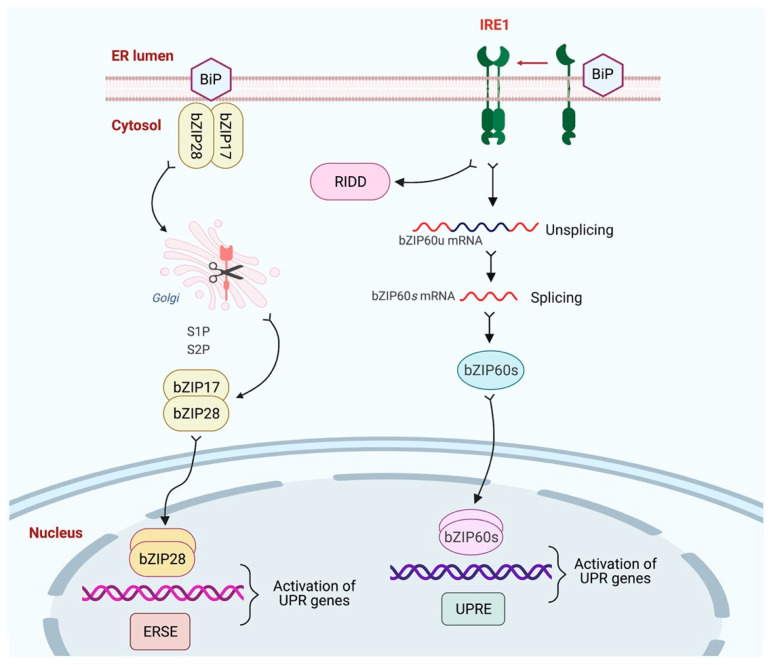
Overview of the pathway for UPR signaling. UPR signaling pathways in plants have two arms/branches. One branch contains the dual protein kinase and ribonuclease, IRE1, responsible for splicing bZIP60 mRNA when activated. The other branch is mediated by bZIP17 and bZIP28, two ER membrane-anchored TFs. A frameshift is introduced by splicing *bZIP60(u)* mRNA so that the resulting spliced type *bZIP60(s)* mRNA is translated into a nucleus-targeted TF. In response to ER stress, the bZIP17 and/or bZIP28 are mobilized and transported to the Golgi, where resident site-1 and site-2 proteases process them and release their cytosolic TF domains [bZIP17(p) and/or bZIP28(p)] into the cytoplasm for further importation into the nucleus. The bZIP17(p) and bZIP28(p) can homodimerize or heterodimerize in the nucleus, where they bind to the promoters and regulate the expression of genes that respond to stress. This figure was created using BioRender software.

A wide range of stresses affect protein folding, causing ER stress that is communicated to the nucleus via the UPR, a cellular homeostatic response to ER stress [106,107]. As a result, genes involved in the folding, import, export, and quality control of proteins are up-regulated. In plants, signal transducers mediate the signaling that forms two arms of the UPR signaling pathway [14,38,43]. One arm includes membrane-associated TFs, for instance, bZIP17 and bZIP28, and the other arm includes IRE1, which is an RNA splicing factor. These two arms shape the stress transcriptome, the upregulation and the downregulation of the expression of genes to combat the stress effects [103]. On the other hand, cells undergo PCD if the adaptation mechanisms are inadequate to manage the unfolded protein load. In plants, the PCD regulatory mechanism and the key factors that regulate various outputs of ER stress receptors remain unclear. The bZIP17/28 are retained in the ER under normal conditions, associating with UPR regulator BiP. However, when unfolded proteins accumulate under stress conditions, BiP is sequestered and released from bZIP17/28 [108,109]. In response to ER stress, bZIP17 and/or bZIP28 are mobilized and transported to the Golgi, where they are proteolytically cleaved by two proteases: Site 1 Protease (S1P), a processing site and Site 2 Protease (S2P), a recognition site. The S1P cleaves them in the Golgi’s C-terminal region and the S2P in its cytosolic end. These two proteases release their TF domains [bZIP17(p) and/or bZIP28(p)] into the cytoplasm for further importation into the nucleus, where they upregulate the expression of stress response genes and restore the ER homeostasis [108,110] (Figure 3). The regulated intramembrane proteolysis (RIP)-mediated activation of the bZIP28 is the first-hand response for mitigating the ER stress in plants. The molecular structure of a type II membrane protein bZIP28 reveals that it comprises a cytoplasmic DNA-binding bZIP domain at its N-terminus, a single transmembrane domain, and a luminal domain at its C-terminus [15].

Additionally, IRE1, a bifunctional protein kinase/ribonuclease, is an essential plant UPR regulator that mediates the cytoplasmic splicing of RNA encoding the TF bZIP60. This triggers the signaling pathway of UPR and regulates the canonical UPR genes. However, it is largely unknown how IRE1’s protein activity is controlled during the growth and development of plants [46]. Two isoforms, IRE1-IRE1a and IRE1b are found in the *A. thaliana* [111]. Both IRE1a and IRE1b isoforms are classified as type I single-pass transmembrane proteins. These comprise multifunctional domains, for instance, a protein kinase domain, an N-terminal signal peptide, a cytosol-facing C-terminal ribonuclease domain, and an ER-stress sensing domain that faces the ER lumen [15]. Both IRE1a and IRE1b specifically activate the *bZIP60* mRNA’s unconventional splicing in response to the biotic and abiotic stresses [42,64]. The plant IRE1a and IRE1b can form homo/heterodimers to activate the IRE1-dependent UPR signaling pathway, similar to how mammals and yeast activate the IRE pathway [63,112].

In the IRE1 pathway, luminal BiPs interact with the ER-membrane protein IRE1 in the ER lumen. After accumulating unfolded proteins, BiPs bind them and release IRE1 proteins that form dimers that, unusually, splice *bZIP60* mRNAs in the cytosol. The spliced mRNA translates into a functional TF, which moves to the nucleus and upregulates the genes containing ER stress elements (ERSEs) and UPR responsive elements (UPREs) in their regulatory regions [45,105,113]. The IRE1 pathway promotes the cytosplicing of *bZIP60*. Moreover, it promotes ER-localized mRNA degradation, which is known as regulated IRE1-dependent decay (RIDD), and autophagy, in order to reduce protein load in ER or timely eliminate the damaged ER [96,114]. The degradation creates a frameshift to encode a type of bZIP60(s) that is transportable to the nucleus [64]. For the activation of stress response genes in the nucleus, the bZIP17(p), bZIP28(p), and bZIP60(s) can homodimerize or heterodimerize [43,110]. RIDD typically contributes to pro-life processes by reducing the abundance of ER client mRNA in ER stress environments [107,115]. Nonetheless, RIDD activity against targeted non-ER client mRNAs has widened the species-specific mannered downstream effects of IRE1 activation [100].

#### 7.2.2. ER-Associated Degradation (ERAD)

ERAD is part of the ER protein quality-control system (ERQC), which is considered essential for the conformation fidelity of most of the membrane and secretory proteins in eukaryotes. The ERAD process is associated with the ubiquitin/proteasome system (UPS), which relieves ER stress. The UPS function involves the use of a ubiquitin-activating enzyme (E1), the ubiquitin-conjugating enzyme (E2), the ubiquitin ligase (E3), and the 26S proteasome [116]. ERAD is accomplished through multistep reactions involving the sequential recruitment of E1, E2, and E3 enzymes. E2 and E3 enzymes are responsible for the specificity of the substrate [92,117]. Since the plants are sessile species, they respond to environmental changes by regulating the signaling pathways from seed germination to the mature organism. Consequently, plant cells express more E3 ligase family members than mammals and yeast cells [92,118]. ERAD is believed to work in plants with core machineries that are highly conserved to those found in yeast and humans, but the ERAD system in plants is poorly understood [98]. The protein folding process is complex and can be easily disrupted [38,66,67,110]. Therefore, conserved ERQC strictly monitors protein folding and identified misfolded proteins to be eliminated by ERAD [66].

#### 7.2.3. Autophagy

Autophagy is a self-eating cellular process that has been conserved throughout evolution. Autophagy functions as a degradation process in recycling cellular cytoplasmic contents and removing damaged proteins or organelles under adverse growth conditions [101]. There are three main types of autophagy on the basis of their mechanism; chaperone-mediated autophagy [119], macroautophagy [120], and microautophagy [121]. Chaperone-mediated autophagy is highly selective, while macroautophagy and microautophagy may be selective or non-selective [122,123]. In plants, the following two types of autophagy are known: macroautophagy and microautophagy [124]. During macroautophagy, the majority of cytosolic constituents are sequestered into a double-membrane structure, known as an autophagosome. The autophagosome is a specific organelle that mediates macroautophagy. For degradation, autophagy delivers cytoplasmic materials or organelles to the vacuole/lysosome by forming autophagosome [99,125]. The autophagosome’s outer membrane fuse with the vacuolar membrane upon delivery to the vacuole, delivering the inner membrane structure and its cargo, i.e., the autophagic body into the vacuolar lumen for degradation by vacuolar hydrolases [126,127]. During microautophagy, the invagination of the vacuolar membrane (autophagic bodies) delivers a part of the cytoplasm to the vacuolar lumen and then the resident vacuolar hydrolases digest them [128]. A number of eukaryotic-conserved autophagy-related (ATG) proteins play a significant role in this process [129].

A selective autophagic pathway for resolving ER stress and restoring the ER homeostasis, ER-phagy, has been defined to remove misfolded or unfolded proteins accumulating in the ER [130]. Similarly, ER-phagy, the autophagy receptors that act as a bridge between autophagic cargoes selection and autophagosome formation, are also needed [131]. Under these ER stress conditions, ER-phagy delivers the unfolded or misfolded ER proteins and fragments for degradation into the vacuole [101], which indicates that ER-phagy receptors are possibly involved in plants [99]. Autophagy can also be significantly induced in plants by ER stress agents, including DTT and TM which prevent proper protein folding in the ER [101]. It was shown by the electron and confocal microscopy that ER portions are engulfed by autophagosomes and delivered to the vacuole, most likely for degradation. Furthermore, one of the ER stress sensors, IRE1b is needed for inducing autophagy by ER stress [101]. In response to ER stress, autophagy upregulation requires IRE1 RIDD activity by degrading the mRNA of proteins involved in autophagy in *A. thaliana*, for instance, b-glucosidase 21 and PR protein 14 [132]. It is still unknown if RIDD is essential for plant survival [100].

## 8. Concluding Remarks and Future Perspectives

Plants have evolved sophisticated signal transduction mechanisms and sensitive detection systems for coping with various stress conditions. Due to climate change, changes in the agricultural environment cause significant reductions in crop yields. To assist crops in coping with newly emerging stresses, it is critical to understand resistance mechanisms of plants. ER is a large, architecturally variable, and functionally complex organelle in eukaryotes. Different intracellular and extracellular stresses may increase the number of misfolded proteins, resulting in an ER homeostasis imbalance, which is referred to as ER stress. Adverse conditions interfere with several sensitive cellular processes in plants that accumulate the misfolded proteins. In plants, UPR mediates the response to many biotic and abiotic stresses. UPR is necessary for the homeostasis of proteins in the ER when adverse environmental conditions challenge plants. In *A. thaliana*, IRE1 is one of the major sensors which activates the bZIP60. The bZIP28 is another sensor that triggers another ER arm, and bZIP17 induces downstream genes. Significant progress has been made in elucidating the UPR signaling in plants and various techniques have been developed to study ER stress in *A. thaliana* and other major crops/plants. The molecular mechanism of ER stress in plants is not as well understood as it is in animals. However, further work is needed to expand the understanding of the mechanism of ER stress and the UPR signaling pathway against various stresses in major crops/plants.

## Abbreviations

AtHSPR	*A. thaliana* Heat Shock Protein Related
AtSec62	Arabidopsis Sec62
BiP	Binding protein/Binding immunoglobulin protein
BiP3	Binding protein 3
BLI	Golgi-localized protein BLISTER
bZIP17	BASIC LEUCINE ZIPPER 17
bZIP60	Leucine zipper transcription factor 60
CNX 1-like	Calnexin 1-like
CPR	Cytosolic Protein Response
CRT	Calreticulin
DTT	Dithiothreitol
EMR	ERAD-mediating RING finger protein
EMS	Ethyl methanesulphonate
ER	Endoplasmic reticulum
ERAD	ER-associated degradation
ERQC	ER protein quality-control system
ERSE	ER stress response element
ETH	Ethylene
FAD2	FATTY ACID DESATURASE 2
GRP94	Glucose-regulated protein 94
HOP	HSP70-HSP90 organizing protein
HY5	ELONGATED HYPOCOTYL 5
hyl1	HYPONASTIC LEAVES1
IRE1	Inositol requiring enzyme1
MAPR3	Membrane-associated progesterone receptor 3
MLO	Barley powdery mildew O
NPR1	Nonexpressor of pathogenesis-related (PR) genes 1
PCD	Programmed cell death
PDI	Protein disulfide isomerase
PPI	Peptidyl-prolyl isomerases
PR	Pathogenesis-related
RIDD	IRE1-dependent decay of mRNAs
RIP	Regulated intramembrane proteolysis
ROS	Reactive oxygen species
S1P	Site 1 Protease
S2P	Site 2 Protease
SA	Salicylic acid
SAR	Systemic acquired resistance
SPL6	SQUAMOSA PROMOTER-BINDING PROTEIN-LIKE 6
TF/TFs	Transcription factor/Transcription factors
TM	Tunicamycin
UPR	Unfolded protein response
UPS	Ubiquitin/proteasome system
VIGS	Virus-induced gene silencing
